# Significant underascertainment in Huntington’s disease

**DOI:** 10.1093/braincomms/fcaf194

**Published:** 2025-05-20

**Authors:** Sujin Lee, Ben Weisburd, Jiwoo Lee, Kevin Correia, Sophia Zeng, Seri S Park, Jun Wan Shin, Doo Eun Choi, Kyung-Hee Kim, Jae-Hyun Jang, Tammy Gillis, Heidi L Rehm, James F Gusella, Marcy E MacDonald, Jong-Min Lee

**Affiliations:** Center for Genomic Medicine, Massachusetts General Hospital, Boston, MA 02114, USA; Department of Neurology, Harvard Medical School, Boston, MA 02115, USA; Center for Genomic Medicine, Massachusetts General Hospital, Boston, MA 02114, USA; Medical and Population Genetics Program, The Broad Institute of M.I.T. and Harvard, Cambridge, MA 02142, USA; Center for Genomic Medicine, Massachusetts General Hospital, Boston, MA 02114, USA; Medical and Population Genetics Program, The Broad Institute of M.I.T. and Harvard, Cambridge, MA 02142, USA; Center for Genomic Medicine, Massachusetts General Hospital, Boston, MA 02114, USA; Center for Genomic Medicine, Massachusetts General Hospital, Boston, MA 02114, USA; Center for Genomic Medicine, Massachusetts General Hospital, Boston, MA 02114, USA; Center for Genomic Medicine, Massachusetts General Hospital, Boston, MA 02114, USA; Department of Neurology, Harvard Medical School, Boston, MA 02115, USA; Center for Genomic Medicine, Massachusetts General Hospital, Boston, MA 02114, USA; Department of Neurology, Harvard Medical School, Boston, MA 02115, USA; Center for Genomic Medicine, Massachusetts General Hospital, Boston, MA 02114, USA; Department of Neurology, Harvard Medical School, Boston, MA 02115, USA; Center for Genomic Medicine, Massachusetts General Hospital, Boston, MA 02114, USA; Department of Neurology, Harvard Medical School, Boston, MA 02115, USA; Center for Genomic Medicine, Massachusetts General Hospital, Boston, MA 02114, USA; Center for Genomic Medicine, Massachusetts General Hospital, Boston, MA 02114, USA; Medical and Population Genetics Program, The Broad Institute of M.I.T. and Harvard, Cambridge, MA 02142, USA; Department of Pathology, Harvard Medical School, Boston, MA 02115, USA; Center for Genomic Medicine, Massachusetts General Hospital, Boston, MA 02114, USA; Medical and Population Genetics Program, The Broad Institute of M.I.T. and Harvard, Cambridge, MA 02142, USA; Department of Genetics, Blavatnik Institute, Harvard Medical School, Boston, MA 02115, USA; Center for Genomic Medicine, Massachusetts General Hospital, Boston, MA 02114, USA; Department of Neurology, Harvard Medical School, Boston, MA 02115, USA; Medical and Population Genetics Program, The Broad Institute of M.I.T. and Harvard, Cambridge, MA 02142, USA; Center for Genomic Medicine, Massachusetts General Hospital, Boston, MA 02114, USA; Department of Neurology, Harvard Medical School, Boston, MA 02115, USA; Medical and Population Genetics Program, The Broad Institute of M.I.T. and Harvard, Cambridge, MA 02142, USA

**Keywords:** Huntington’s disease, disease prevalence, frequency of expanded *HTT* CAG repeat, exponential decay model, underascertainment

## Abstract

While Huntington’s disease (HD), a Mendelian disorder caused by an expanded CAG repeat in *HTT*, is considered rare, the true prevalence could be significantly higher due to substantial underascertainment. Given inherent biases in empirically assessing disease prevalence, we performed mathematical modelling and validation analyses to estimate the frequency of expanded CAG repeats in the general population to better understand the disease prevalence. We developed an exponential decay model after confirming that the logarithmic decrease in frequency of CAG repeats extends into the pathogenic range (CAG > 35). The model was further refined by incorporating HD onset and mortality probabilities to estimate the clinical ascertainment rate. Our age-adjusted exponential decay model estimated one expanded repeat in 325 people and further showed that the frequency of expanded repeats decreases with age due to the early mortality associated with HD, which was validated by All of Us and UK Biobank data. Importantly, our data suggest that approximately half of symptomatic HD individuals aged 30–70 are not clinically ascertained/diagnosed. Our data, showing higher frequencies of expanded repeats in the general population and significant underascertainment rates, imply that HD prevalence could be twice as high as current estimates.

## Teaser

Lee *et al*. performed mathematical modelling/validation analyses to objectively determine the clinical ascertainment rate of Huntington’s disease and revealed higher frequencies of expanded huntingtin CAG repeats in the general population and significant underascertainment rates, indicating that Huntington’s disease prevalence could be double the current estimates.

## Graphical Abstract

**Graphical Abstract:**
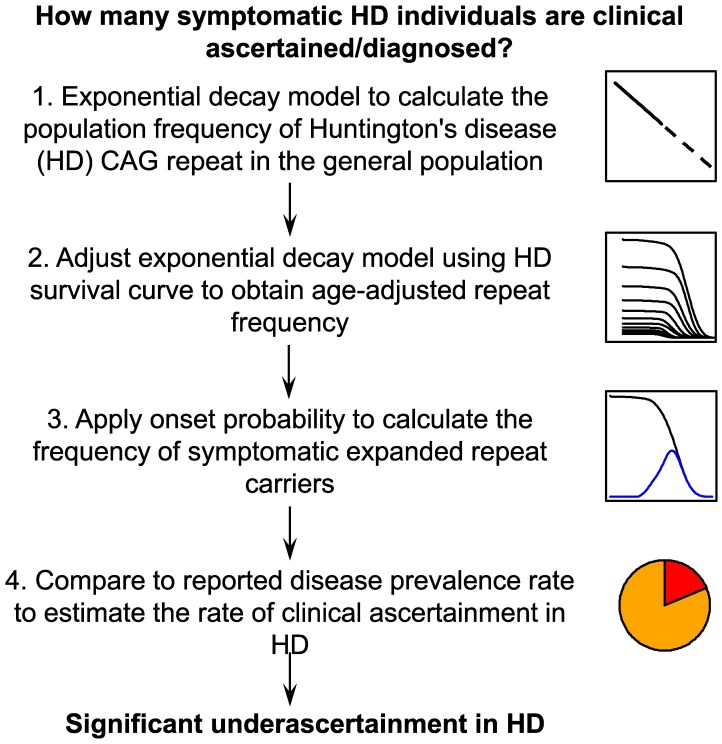

## Introduction

Huntington’s disease is caused by an expanded CAG trinucleotide repeat (CAG > 35) in huntingtin (*HTT*).^1-4^ Since the identification of the cause of the disease, significant progress has been made in the areas of animal modelling, mechanisms, therapeutic strategies and genetic modifiers.^2-5^ Despite these significant advancements, fundamental aspects of Huntington’s disease remain unresolved, particularly regarding (i) the frequencies of expanded repeats in the general population; and (ii) the true prevalence rate of the disease, both of which are challenging to determine due to limitations and biases inherent in cohort-based studies.

The classification of Huntington’s disease as a rare disease stems from earlier prevalence estimates, which widely vary depending on ancestry and location.^6-10^ The highest prevalence (∼12/100 000) was observed in UK-based studies.^11,12^ However, growing evidence suggests that these studies might have significantly underestimated the prevalence of Huntington’s disease.^10,11^ This underestimation of prevalence could be due to several factors. Earlier studies, conducted before the genetic cause of Huntington’s disease was discovered, faced challenges in accurately diagnosing the disease, particularly when psychiatric symptoms were the first manifestations.^12,13^ In addition, limited access to health care systems, negative social recognition and concerns with insurance may have discouraged individuals with disease from seeking clinical confirmation.^12^ Unless such factors are adequately addressed, traditional population-based surveys that rely on clinical diagnosis are likely to underestimate the prevalence of Huntington’s disease.

The primary objective of our study was to gain a more objective estimation of the prevalence of Huntington’s disease by focusing on the underlying genetic mutation rather than relying on clinical diagnoses. Specifically, we employed a novel approach that combined mathematical modelling with follow-up validation analyses to estimate the frequency of expanded CAG repeats in the general population, aiming at calculating the rate at which symptomatic Huntington’s disease individuals are clinically ascertained.

## Materials and methods

### Key terms and overall procedure

This manuscript uses the term ‘frequency’ to refer to the occurrence regardless of disease manifestation or diagnosis status of Huntington’s disease. The term ‘prevalence’ refers to the number of clinically diagnosed expanded repeat carriers with characteristic motor symptoms. Ascertainment rate refers to the number of clinically ascertained/diagnosed expanded CAG repeat carriers relative to all expanded CAG repeat carriers (irrespective of clinical manifestation or symptoms). The overall procedure and approach are summarized in Supplementary Fig. 1.

### CAG repeats in clinically ascertained Huntington’s disease subjects of European ancestry for statistical modelling

**Figure 1:**
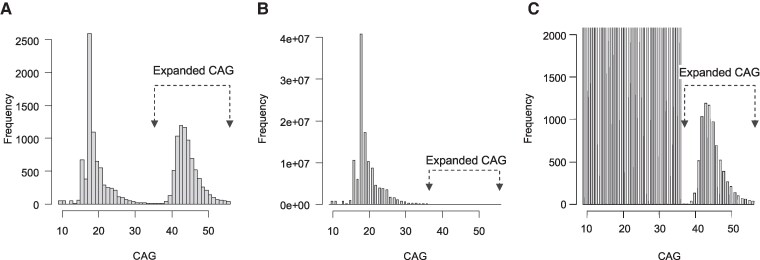
**Estimating the frequency of CAG repeats based on the reported disease prevalence**. (**A**) Histogram showing the distribution of CAG repeat sizes among 7578 clinically ascertained and unrelated Huntington’s disease subjects of European ancestry who participated in previous modifier GWAS studies, representing a total of 15 156 chromosomes. (**B**) A simulated dataset was generated by combining the 7578 expanded CAG repeats from the study samples with 122 210 406 bootstrapped unexpanded repeats. This simulation assumes that expanded repeats dominantly cause the disease and that all carriers are clinically ascertained. The resulting CAG repeat distribution matches the reported prevalence rate (one in 8064) and is visualized in a histogram, where each bar corresponds to a specific CAG repeat length. (**C**) The distribution of expanded CAG repeats from the data simulated to match the reported prevalence rate is shown in a zoomed-in histogram to provide a clearer view of the relatively small population of expanded repeats (observed expanded repeats, *n* = 7578; bootstrapped unexpanded repeats, *n* = 122 210 406).

In our previous genome-wide association studies (GWAS) aimed at identifying genetic modifiers of Huntington’s disease,^5,14^ we focused on Huntington’s disease subjects of European ancestry with CAG repeat lengths smaller than 56, to minimize potential bias; this range represented 96.6% of the data. For the current study, we similarly analysed CAG repeats < 56 for initial modelling. Using data from our prior GWAS,^5^ we identified unrelated individuals carrying one expanded CAG repeat using the KING program (options: --unrelated --degree 2). This approach identified 7578 Huntington’s disease subjects (each with one expanded and one unexpanded CAG repeat) for statistical modelling of *HTT* CAG repeat frequency (3710 males and 3868 females) (Fig. 1). For subsequent analyses of probabilities of onset and mortality, we focused on individuals with CAG repeat lengths between 40 and 50 (representing 90% of the data) to reduce bias from smaller sample sizes at other repeat lengths. Details on the methods used to determine CAG repeat sizes are provided elsewhere.^5,15^

### Frequency of CAG repeats in gnomAD, All of Us data and the UK Biobank

Summary of CAG repeat frequencies was obtained from the gnomAD browser (v3.1.2; https://gnomad.broadinstitute.org/) and the All of Us data browser (downloaded 8 April 2022; https://www.researchallofus.org/data-tools/) by querying the chr4:3074870-3074940 region (GRCh38). For the gnomAD data, quality control filtering was applied to identify CAG repeat length polymorphisms discovered through whole genome sequencing. These data were then used to assess whether (i) unexpanded repeats in Huntington’s disease subjects are representative of those in the general population; and (ii) the exponential decrease in repeat frequency persists beyond a CAG length of 35. We also determined sizes of repeats of participants of the UK Biobank (https://www.ukbiobank.ac.uk/; 490 380 samples) using the ExpansionHunter program (v5).^16^ Then, we generated REViewer images 5 for the individuals with 40 or more repeats to evaluate the genotype quality and check for interruptions. Manual inspection of these images confirmed high genotype quality in all 161 individuals (40–46 CAGs). We also recorded the G10 ICD code within the UK Biobank primary care, hospital admissions and cause of death records to determine which individuals had been previously diagnosed with Huntington’s disease. For validation of our estimation for ascertainment rate, we focused on CAG repeats with sample size > 10 (i.e. 40–42 CAGs).

### Estimation of CAG repeat frequencies using reported disease prevalence rates

Given the significant impact of expanded CAG repeats, the prevalence of Huntington’s disease reflects, to some extent, the frequency of disease-causing repeats in the general population. We utilized widely accepted prevalence estimates (12.4 per 100 000 individuals; approximately one in 8064)^11,17^ to estimate the frequencies of expanded CAG repeats in the European ancestry population. In making these estimates, we assumed that (i) all expanded CAG repeats cause Huntington’s disease in a dominant manner; and (ii) all individuals carrying expanded repeats are clinically ascertained. While these assumptions do not fully account for the CAG repeat length-dependent and age-dependent clinical manifestations, they enable us to approximate the frequency of expanded repeats despite limited prior knowledge about the level of clinical ascertainment, which this study aimed to determine.

The reported Huntington’s disease prevalence rate and our assumptions suggested that 61 108 992 individuals from the general population would need to be sampled to obtain our study cohort of 7578 expanded repeats we ascertained. To calculate the frequency of expanded repeats in the general population, we combined the 7578 expanded repeats with 122 210 406 unexpanded repeats (i.e. 61 108 992 × 2 − 7578). Given the impracticality of collecting ∼61 million individuals, we generated simulated unexpanded repeats through bootstrapping. Since the frequencies of unexpanded repeats in Huntington’s disease subjects were similar to those observed in population samples (e.g. gnomAD, All of Us and UK Biobank), and our repeat data were experimentally determined,^1^ we generated 122 210 406 unexpanded repeats by bootstrapping from the unexpanded repeats in our study samples.

### Fitting common probability distributions to the frequencies of unexpanded repeats of study samples

To identify statistical models that could explain the frequency distribution of unexpanded CAG repeats, we fitted several commonly used probability distributions to the observed data: normal (mean = 18.41, SD = 3.28), Poisson (λ = 18.411), negative binomial (size = 1922.7, μ = 18.41), Cauchy (location = 17.34, scale = 1.10), gamma (shape = 33.59, rate = 1.82), log-normal (meanlog = 2.89, sdlog = 0.17), logistic (location = 18.04, scale = 1.64) and Weibull (shape = 5.17, scale = 19.81). The fitness of each distribution was graphically evaluated by comparing the theoretical models to the histogram of observed unexpanded repeat frequencies in the study samples, revealing that none of the commonly used distributions adequately explained the frequency distribution of unexpanded repeats. However, the log-transformed frequencies of repeats > 17 showed a linear relationship in the study samples, with an *R*-squared value of 0.985. This finding led us to focus on an exponential model to describe the decrease in frequencies of both unexpanded and expanded repeats.

### Initial exponential decay model for estimating repeat frequencies at birth

**Figure 2:**
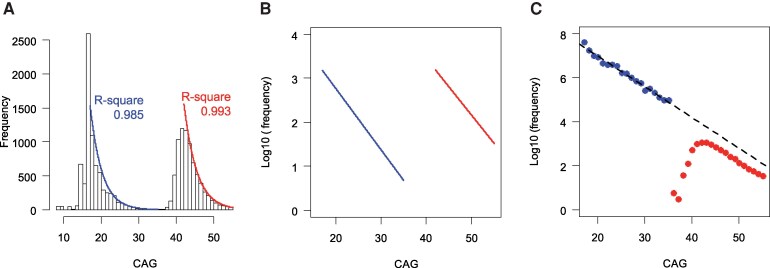
**Exponential decay model for frequencies of expanded repeats in the general population**. (**A**) The histograms show the distributions of CAG repeat sizes in the 7578 study samples. Separate linear regression models (using statistical analysis ‘lm’ function in R) were fitted to log-transformed counts of unexpanded (left histogram) and expanded (right histogram) CAG repeats to assess whether the frequencies decrease exponentially after 17 CAGs. (**B**) Exponential decay models for normal (left line) and expanded (right line) CAG repeats are plotted on a logarithmic scale (log10 of % frequency), revealing parallel decay trends. (**C**) Based on the following observations: (i) the exponential decrease in the frequency of unexpanded repeats; (ii) the continuation of this trend beyond 35 CAGs in large population samples; and (iii) exponential decay patterns being largely parallel for unexpanded and expanded repeats, we constructed an exponential decay model using the frequencies of unexpanded repeats alleles. This model was then extended to the expanded repeat range (dashed line) to estimate the frequencies of expanded repeats in the general population. Each dot represents an observed frequency of individuals carrying expanded CAG repeats (*n* = 7578).

Our data suggested that the frequency of CAG repeats in the general population peaks at 17 CAGs and then continuously and exponentially decreases, even within the expanded repeat range (CAGs > 35). This hypothesis was based on two key observations: (i) the logarithmic decrease in frequency continued beyond 35 CAGs in the population samples; and (ii) the exponential decay patterns for unexpanded and expanded repeats were largely parallel. In addition, the log-transformed frequencies of CAG repeats in large population samples (e.g. gnomAD, All of Us and UK Bank) exhibited a linear trend starting from the most frequent repeat length (17 CAGs), with this pattern continuing into the expanded repeat range. Given that the logarithmic decrease in frequencies of unexpanded repeats was observed in both study and population samples, we hypothesized that the frequencies of repeats > 17 decrease exponentially. We thus fitted a linear regression model to the log-transformed frequencies of 18–35 repeats from the study samples, resulting in an exponential decay model for unexpanded repeats (*P* = 5.1E^−17^; *R*-squared = 0.985; Fig. 2A, blue). A separate linear regression model was applied to the frequencies of 42–55 repeats in the study samples, producing an exponential decay model for expanded repeats (*P* = 2.0E^−14^; *R*-squared = 0.993; Fig. 2A, red). Since (i) the frequency decrease persisted beyond 35 CAG repeats, and (ii) the exponential decay models for unexpanded and expanded repeats were largely parallel, our data suggest that the frequency of repeats peaks at 17 and then continuously decreases exponentially, even into the expanded repeat range. Consequently, we extended the exponential model for unexpanded repeats to the expanded repeat range to estimate the frequencies of expanded repeats in the general population. This final exponential decay model (log10(% frequency) = 5.552482 − 0.13925 × CAG, where CAG ranges from 17 to 55) was used to calculate the frequency of each repeat length (Fig. 2C).

### Incorporating survival probability to correct age-related effects on the frequency of expanded repeats

**Figure 3:**
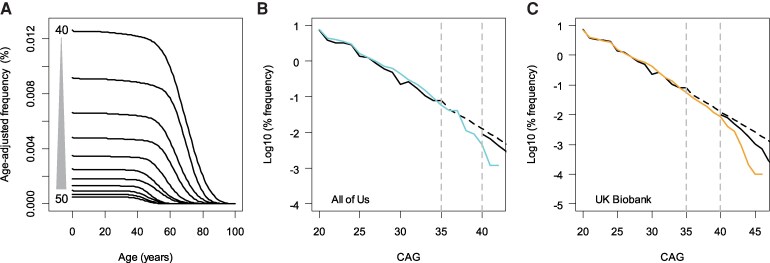
**Age-adjusted exponential decay model**. (**A**) The age-dependent decreased survival in Huntington’s disease (based on statistical analysis ‘survreg’) has been incorporated into the model to generate the age-adjusted exponential decay model. Age adjustments were applied to the 40–50 CAG repeat range, where sufficient sample sizes were available for the Huntington’s disease survival model. Each line represents the frequency of a specific CAG repeat size (40–50 CAG). (**B**) The age-adjusted exponential decay model was validated against the observed frequency of the repeats in the All of Us data. A simulated population was generated to mimic the age distribution of the All of Us participants (age range, 18–99; *n* = 425 906), and then the frequencies of expanded repeats in this simulated population were calculated based on the age-adjusted exponential decay model (solid line). A dashed line represents the original exponential decay model, which was used to predict the frequencies of 36–39 CAG repeats. The observed frequencies of expanded repeats in the All of Us dataset (a solid line spanning 20–42 CAGs without a dashed section) were compared to the predicted frequencies from our models using Pearson’s chi-squared test (*P*-value, 0.260). (**C**) The same procedures were taken to validate our age-adjusted exponential decay model using UK Biobank data (age range, 37–73; *n* = 502 618). A dashed line represents the original exponential decay model, which was used to predict the frequencies of 36–39 CAG repeats. The observed frequencies of expanded repeats in the UK Biobank data (a solid line spanning 20–45 CAGs without a dashed section) were compared to the predicted frequencies from our models using Pearson’s chi-squared test (*P*-value, 0.242). Note that the frequencies of repeats > 40 in All of Us and the UK Biobank began to deviate from our age-adjusted estimate, which was anticipated due to the potential enrichment of healthy individuals in these cohorts.

Since Huntington’s disease is associated with early mortality, the frequency of expanded repeats is expected to decrease over time. However, our initial model, based on unexpanded repeats, represents repeat frequencies at birth, as these repeats are unlikely to affect mating behaviour or survival. To estimate the frequencies of expanded repeats at older ages, we adjusted our initial exponential model by incorporating the death probability of Huntington’s disease subjects. We focused on CAG repeat lengths of 40–50 (from 1165 Huntington’s disease subjects) to obtain reliable death probability estimates, as repeats outside this range had small sample sizes in our recorded age-at-death data (*n* < 30).^18^ For each CAG repeat, we fitted a parametric survival model using the ‘survreg’ function to the Huntington’s disease mortality data. Since our data consisted of recorded ages at death, survival models were fitted without censoring. Various survival models based on different distributions (e.g. Weibull, exponential, Gaussian, logistic, log-normal and log-logistic) were visually inspected to compare the fit, with the log-normal distribution providing the best fit. Consequently, subsequent survival models were fitted using the log-normal distribution. Our death probability estimates likely reflect mortality due to Huntington’s disease, as the models were based on recorded deaths in Huntington’s disease subjects, with an onset-to-death duration of ∼15 years.^8^ Given the lack of unbiased estimates for the survival probability of Huntington’s disease subjects, we used the reciprocal of death probability (i.e. 1/death probability) as the proxy of survival to adjust the original exponential decay model. Additionally, individuals carrying expanded repeats may die from causes other than Huntington’s disease, further reducing their frequency. Although such probabilities could not be directly inferred from our Huntington’s disease death data, mortality due to non-Huntington’s disease causes might be captured in the survival data of the general population. The survival probability of the general population was calculated based on age-specific mortality data from the actuarial life table, which was downloaded from the U.S. Social Security Administration website (https://www.ssa.gov/oact/STATS/table4c6.html); we averaged male and female data from the years 2008–24. To calculate the overall survival probability of individuals carrying expanded repeats, we multiplied the survival probability for Huntington’s disease by the survival probability for the US general population, assuming that deaths from Huntington’s disease and other causes are independent. Finally, age-specific survival probability was multiplied to our initial exponential decay model to generate age-adjusted exponential decay model. The age-specific frequencies of expanded repeats from our age-adjusted exponential decay model are summarized in Fig. 3A.

### Validation of the age-adjusted exponential decay model using All of Us and UK Biobank data

The age-adjusted exponential decay model was validated using data from the All of Us and UK Biobank data. Since the frequency of expanded CAG repeats varies with age, we simulated the frequency of each repeat according to our age-adjusted model, aligning the simulations with the age distribution and composition of participants in All of Us and UK Biobank. Since age distribution was not clearly specified in the gnomAD data, those data were not used for validation. To validate the model for the 42 CAG repeat, for example, we generated a simulated dataset of 100 000 individuals that matched the age distribution of the All of Us and UK Biobank participants. We then calculated the number of individuals at each age and determined the count of the 42 CAG repeat at each age by multiplying the number of individuals by the age-specific frequency. The allele frequency was then obtained by dividing the total count of the 42 repeat allele across the entire age range by 200 000 alleles (i.e. 100 000 people). These predictions are represented as solid black lines beyond 40 CAG repeats in Fig. 3B and C, where the age-adjusted exponential decay model was applied to the 40–50 CAG range. Statistical differences between observed and predicted frequencies of expanded repeats were assessed using the chi-squared test.

### Estimating the rate of clinical ascertainment using Huntington’s disease onset probability

Since only surviving individuals carrying expanded CAG repeats who exhibit disease symptoms can be clinically ascertained, we aimed to estimate the rate of clinical ascertainment by first calculating the probability of onset. Our age-adjusted exponential decay model for repeat frequency represents the proportion of surviving individuals with expanded repeats in the population. We then estimated the probability of onset using recorded age at onset data for motor symptoms.^5^ To ensure reliable estimation and avoid errors due to small sample sizes, we focused on samples carrying 40–50 CAG repeats (*n* = 6827), similar to our approach in estimating Huntington’s disease death probability. The probability of onset for each CAG repeat was calculated based on the recorded age at motor onset, with survival models fitted using the ‘survreg’ function and log-normal distribution, which provided the best fit to the data. We applied this age-specific onset probability to estimate the frequency of individuals carrying expanded repeats, both with and without disease symptoms. Although our estimation was based on recorded onset data, which could introduce bias as our data did not include expanded allele carriers who had not yet developed symptoms, it was expected that these carriers would eventually manifest symptoms. Their onset ages would likely fall within the range of recorded onset ages based on large sample size, minimizing potential bias. Supporting this, our onset probability estimates, based on a large sample size, were similar to those derived from survival models in previous studies.^19^ For example, the ages at 50% onset probability for 41, 42, 43, 44 and 45 CAG repeats, as estimated by survival analysis^9^ and our analysis, were 57, 52, 48, 44 and 41.5 years, and 56.5, 52, 48, 43.5 and 40.5 years, respectively.

### Calculation and validation of the clinical ascertainment rate

We aimed to estimate the proportion of expanded allele carriers with clinical symptoms who were clinically ascertained/diagnosed relative to all symptomatic expanded repeat carriers, which we defined as the rate of clinical ascertainment. Specifically, we calculated this rate by comparing the number of individuals carrying 40–50 CAG repeats who developed clinical symptoms with the widely accepted Huntington’s disease prevalence data (i.e. 12.4 per 100 000 people) as a surrogate for ascertained expanded repeat carriers. Since disease prevalence estimation is influenced by the age-dependent frequency of expanded repeats and the probability of onset, we estimated the ascertainment rate across various age ranges. For a given age range, we generated simulated data, matching the population pyramid structure of the European Union (https://www.populationpyramid.net/europe/2020/). For example, we constructed a simulated population of 100 000 people, with the following distribution across age groups: 1 050 000 people in the 0–9 years age group, 1 052 000 in the 10–19 years group, 1 139 000 in the 20–29 years group, 1 409 000 in the 30–39 years group, 1 396 000 in the 40–49 years group, 1 392 000 in the 50–59 years group, 1 224 000 in the 60–69 years group, 808 000 in the 70–79 years group, 443 000 in the 80–89 years group, 87 000 in the 90–99 years group and 2000 in the 100+ years group. In this simulated general population, we estimated the number of individuals carrying expanded repeats based on our age-adjusted exponential decay model. We then estimated the number of surviving individuals carrying expanded repeats, focusing on the 40–50 CAG repeat range, using our HD death probability model. For example, in a simulated population of 100 000 individuals aged 30–79, aligned with the EU population pyramid, our model predicted 64 surviving individuals with 40–50 CAG repeats. Since not all surviving carriers of expanded alleles develop disease symptoms, we then estimated the number of symptomatic expanded repeat carriers by multiplying the frequency of surviving expanded repeat carriers by the onset probability. The onset probability was derived from a parametric survival model fitted to recorded ages at onset in Huntington’s disease subjects with 40–50 CAG repeats as described above. In this example, our model predicted that of the 64 individuals with 40–50 repeats, 22 would be symptomatic and 42 asymptomatic. Given that the 40–50 repeat range represents 90% of all expanded repeats, we adjusted the prevalence estimate of 12.4 per 100 000 individuals accordingly. By using 90% of this estimate (i.e. 11.6 per 100 000), we calculated the ascertainment rate by dividing this adjusted prevalence estimate by the number of symptomatic expanded repeat carriers in the simulated population. Validation of clinical ascertainment rates were performed using UK Biobank data. In the UK Biobank, we identified 148 individuals with 40–42 CAG repeats, of whom 36 were clinically ascertained as evidenced by an ICD-10 code for Huntington’s disease (‘G10’). Based on our onset probability and the age distribution in the UK Biobank data (age range, 37–73), we estimated that 84 were symptomatic, resulting in a clinical ascertainment rate of 42.7%.

### Statistical analysis

All statistical analysis, modelling and plotting were performed using R 4.2.2. The ‘survival’ package (version 3.6-4) was used for survival analyses for death and onset. Pearson’s chi-squared test was performed to compare age-adjusted exponential decay model to observed frequencies of expanded repeats in the All of US and UK Biobank data.

## Results

### Estimating the frequencies of *HTT* CAG repeats based on the reported disease prevalence

As an initial attempt to estimate the frequencies of CAG repeats in the general population of European ancestry, we modelled the frequencies based on the CAG repeats in clinically ascertained Huntington’s disease patients and the widely accepted prevalence rate (12.4 per 100 000 people).^17^ Our analysis of CAG repeats from 7578 unrelated Huntington’s disease subjects of European descent (Supplementary Table 1; Fig. 1A), who were identified by genotype (CAGs > 35) and phenotype (motor symptoms) for our GWAS studies.^5^ Under the assumptions that (i) an expanded CAG produces the disease in a dominant manner; and (ii) all individuals with expanded CAG repeats are clinically ascertained, one would need to sample ∼61.1 million individuals (∼122.2 million chromosomes) to obtain 7578 Huntington’s disease patients based on the aforementioned prevalence rate. As unexpanded repeats in Huntington’s disease subjects reflected those in the general population (Supplementary Fig. 2), we combined 7578 expanded repeats with ∼122 million unexpanded repeats bootstrapped from the Huntington’s disease subjects to construct a frequency model representative of the general population. This model revealed a bimodal distribution, with an extremely minor peak corresponding to expanded CAG repeats (Fig. 1B). As there is no *a priori* reason for a sharp frequency drop at 36 CAGs followed by a gradual increase thereafter (Fig. 1C), these data suggested a significant number of individuals with expanded CAG repeats who either (i) have not yet developed symptoms and therefore remain clinically unascertained; or (ii) have developed the disease but escaped clinical detection.

### Exponential decay model to estimate the frequencies of repeats at birth

Small population sample studies have shown that the frequency of the unexpanded repeat begins to decrease at 18 CAGs, with a continuous decline that extends beyond 35 CAGs.^20-22^ We thus investigated whether the bimodal distribution observed in the prevalence-based frequency model was attributable to (i) asymptomatic expanded repeat carriers; or (ii) symptomatic Huntington’s disease individuals who remain unascertained. The distributions of repeats in larger population samples were also continuous rather than bimodal (Supplementary Fig. 3); the scarcity of fully penetrant repeats in these studies was somewhat expected, as they may be enriched for relatively healthy individuals.^23,24^ Nonetheless, these data suggested that the decrease of frequency resembled a logarithmic pattern and continued beyond 35 CAG, supporting that the CAG repeat distribution is continuous and an exponential model for unexpanded repeats could also account for the frequencies of expanded repeats.

The frequency decline of unexpanded repeats after reaching a peak at 17 CAGs was well-explained by an exponential decay model (Fig. 2A, blue; *R*-square, 0.985), contrasting with other commonly used distribution models (Supplementary Fig. 4). A separate exponential decay model also effectively captured the frequency of expanded CAG repeats after the peak at 42 CAGs in the patient samples (Fig. 2A, red; *R*-square 0.993). Notably, the exponential frequency decline patterns for unexpanded and expanded repeats in Huntington’s disease patients were largely parallel (Fig. 2B), reinforcing the hypothesis that frequencies of CAG repeats decrease exponentially after 17 CAGs and that this trend persists within the expanded repeat range. Consequently, we constructed a CAG repeat frequency model based on the exponential decay pattern of unexpanded repeats and extended it into the expanded repeat range (Fig. 2C, black dashed line), revealing significantly higher frequencies of expanded repeats in the general population than the inference obtained from the previously reported prevalence rate.

### Age-adjusted exponential decay model corrected for reduced survival in Huntington’s disease

Since our initial exponential decay model was based on the frequency distribution of unexpanded CAG repeats, which neither cause Huntington’s disease nor confer overtly fitness,^1,25^ the frequencies of expanded repeats derived from our initial model likely represent those at birth. However, given that Huntington’s disease is associated with earlier mortality (Supplementary Fig. 5), the frequencies of expanded CAG repeats are expected to decline with age. To calculate age-corrected frequencies of CAG repeats in the general population, we incorporated the overall survival probability of Huntington’s disease subjects (Supplementary Fig. 6), yielding an age-adjusted exponential decay model that predicted the age-dependent decrease in frequencies (Fig. 3A). For example, the frequency of 42 CAGs was estimated to be 0.00667% at birth, decreasing to approximately half of that frequency by age 64, at which point the survival probabilities for Huntington’s disease and the general population are 61.2% and 84.5%, respectively.

We validated our model using larger population datasets (Supplementary Fig. 7); the frequencies predicted by our age-adjusted exponential decay model were not significantly different from those observed in All of Us (Fig. 3B, *P*-value, 0.260) and the UK Biobank for expanded repeats (Fig. 3C, *P*-value, 0.242), supporting the model’s validity. We also compared our predictions to reported frequencies in the literature. As summarized in Supplementary Table 2, our model predicted slightly higher frequencies for fully pathogenic and all pathogenic repeats compared to estimates from Kay *et al*. and Ibanez *et al*.^26,27^ This difference might be due to the possibility that our estimation aimed at the general population and the aforementioned studies might have an ascertainment bias against Huntington’s disease or healthy volunteer bias.^23,24^ Regardless, our exponential decay model and large population sample studies predicted significantly higher numbers of expanded CAG repeats in the general population compared to the estimates based on the disease prevalence.

### Significant underascertainment in Huntington’s disease

**Figure 4:**
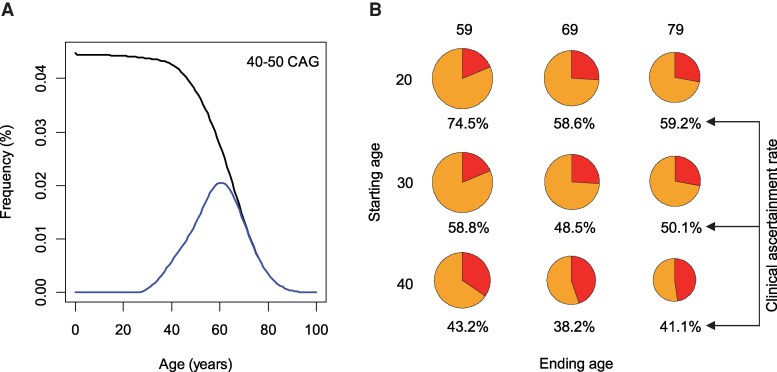
**Significant underascertainment in Huntington’s disease**. (**A**) To calculate the rate of clinical ascertainment, we integrated data for 40–50 CAG repeats into our age-adjusted exponential decay model (based on statistical analysis ‘lm’) alongside onset probability (based on survival analysis using ‘survreg’). The top line represents the frequency of 40–50 CAG repeats combined, while the bottom line indicates the frequency of carriers with disease symptoms. The area under the curve of the bottom line represents the population of individuals carrying 40–50 CAG repeats who have developed symptoms and therefore can be clinically ascertained/diagnosed. (**B**) Using the frequency of expanded repeats based on the age-adjusted exponential decay model (based on ‘lm’ analysis) and onset probability (based on survival analysis using ‘survreg’), we estimated the percentage of clinical ascertainment based on a simulated population (*n* = 100 000). Each pie chart shows the proportions of expanded repeat carriers without disease symptoms (bigger pie) and those with disease symptoms (smaller pie) within a specific age range. The percentage of clinical ascertainment for each age range (indicated below each pie chart) was calculated by dividing the reported prevalence rate by the number of carriers with disease symptoms. For example, if the prevalence rate (i.e. one in 8064 people) was determined from a randomly selected 100 000 people aged 30–79, our data suggest that approximately equal number of symptomatic expanded repeat carriers remain unascertained (middle right pie chart).

The significant differences between our age-adjusted exponential decay model and disease prevalence-based frequencies of expanded repeats implied that a substantial number of individuals carrying expanded repeats are not clinically ascertained. Many individuals carrying reduced penetrance CAG repeats may develop disease symptoms later in life,^5,13,14,28^ resulting in a decreased likelihood of clinical ascertainment and accounting for some of the difference. As a significant difference was also observed for fully penetrant repeats (Fig. 2C), we hypothesized that not all symptomatic Huntington’s disease individuals are clinically ascertained. Thus, we set out to estimate the rate of clinical ascertainment to gain insights into true disease prevalence. Given that the frequencies of expanded CAG repeats vary by age and only surviving symptomatic carriers can be clinically ascertained, we integrated Huntington’s disease onset probability (Supplementary Fig. 8) into our age-corrected frequency data to estimate the clinical ascertainment rate. As the total number of symptomatic expanded repeat carriers for each age group represents the maximum ascertainable samples (Supplementary Fig. 9), this estimation could be compared to the disease prevalence rate to calculate the clinical ascertainment rate. Focusing on individuals with 40–50 CAGs, comparison of the number of ascertainable samples (Fig. 4A) to disease prevalence data revealed significant underascertainment (Fig. 4B). For example, if the disease prevalence rate was based on people aged 30–79, only 50.1% were clinically ascertained. Although the rates of clinical ascertainment were significantly influenced by age, all estimates suggested significant underestimation in the disease prevalence data. Supporting this finding, UK Biobank data (Supplementary Fig. 10; age range, 37–73) revealed a clinical ascertainment rate of 42.7%, which relatively aligns with our estimate. Together, our findings suggest that expanded CAG repeats are more prevalent than currently appreciated and that approximately half of symptomatic expanded repeat carriers remain outside of clinical ascertainment.

## Discussion

We used one of the highest reported prevalence rates^11,12^ to calculate the rate of clinical ascertainment in Huntington’s disease as multiple lines of evidence suggest that even this value unquestionably underestimates the true prevalence due to various biases.^10,11,29^ For example, potential genetic discrimination may have negatively impacted individuals with a family history of Huntington’s disease,^30,31^ decreasing clinical diagnosis. In addition, a significant proportion of individuals with expanded CAG repeats may develop clinical symptoms later in life^14,32,33^ and/or exhibit only mild disease symptoms,^34^ potentially due to genetic modifiers.^5,15^ Both factors may reduce their motivation for clinical ascertainment. Historically, the diagnosis of Huntington’s disease was based primarily on symptoms, without genetic testing.^17^ This approach, combined with the varied presentation of Huntington’s disease symptoms, particularly when psychiatric symptoms appear first,^12,35,36^ could also have led to misdiagnosis^37^ and therefore underestimation of Huntington’s disease prevalence.

Recognizing that uncovering the true prevalence of Huntington’s disease through empirical approaches suffers from challenges and bias, we adopted an alternative strategy of constructing mathematical models to objectively estimate the frequencies of expanded repeats and the prevalence of Huntington’s disease. Our age-adjusted exponential decay model and validation data showed that the genetic cause of Huntington’s disease is quite frequent, with one expanded repeat in every 325 people of the general population. Estimates of the high frequency of intermediate (one in 17) and reduced penetrance (one in 417) alleles are also particularly notable because these repeat sizes can generate pathogenic and fully penetrant repeats through intergenerational instability.^38,39^ Considering the high new mutation rate,^13,40^ our data revealing unexpectedly high frequencies represent a quite significant public health concern.

Our analysis underscores the critical need for unbiased and representative sampling across various age groups when estimating the frequency of expanded repeats and ascertainment of Huntington’s disease and other repeat expansion disorders. However, many studies rely on volunteer cohorts, which tend to favour participants with fewer medical issues. This phenomenon is known as ‘healthy volunteer bias’ where participants are often healthier than the general population.^23,24^ Consequently, such cohorts may underrepresent individuals with diseases, which can be particularly pronounced in genetic disorders like Huntington’s disease, where concerns about genetic discrimination may be substantial. Although not significantly different, model-based estimations of expanded repeat frequencies were slightly higher than those observed in the All of Us and UK Biobank data, likely reflecting this bias. Conversely, an analysis of cohorts with phenotypes that are also observed in Huntington’s disease could reflect an inflated frequency of expanded repeats due to misdiagnosis.^41,42^ Additionally, volunteer-based cohorts may not accurately capture the age distribution of the general population, generating significant bias when studying age-dependent disorders like Huntington’s disease. For instance, the highest average prevalence rate was observed for the age group 51–55.^11^ However, large volunteer-based cohorts tend to skew older,^24^ suggesting that the frequency of expanded repeats in these samples is likely lower than in a representative population due to the higher early mortality associated with Huntington’s disease.^18^ In light of these considerations, our data, which is subject to less bias, represents a significant advancement in accurately estimating the true prevalence of Huntington’s disease.

While our data provide important insights into the fundamentals of Huntington’s disease, it is important to note that the findings are primarily applicable to individuals of European ancestry. Our models were developed using data from Huntington’s disease subjects of European descent,^5^ which limits the generalizability of the frequency estimates across different populations. In the literature, the frequencies of expanded repeats widely varied by geographical regions.^6^ Notably, the highest frequency was observed in European populations, which may partly explain the differences between our age-adjusted exponential decay model and findings from the UK Biobank data that include diverse ancestries. Despite these limitations, our statistical modelling approach employed in this study could be adapted for use in Huntington’s disease populations from other geographical regions or genetic ancestry to generate objective estimates of expanded repeat frequencies across diverse ancestries, contributing to a more comprehensive understanding of Huntington’s disease prevalence worldwide.

It has been proposed that clinicians should not assume that Huntington’s disease is rare outside of known pedigrees.^12,13^ Our findings highlight the relatively high frequency of expanded repeats in the general population and significant underascertainment of affected individuals. This awareness can contribute to shifting negative perceptions of Huntington’s disease and encourage the development of legislation aimed at preventing genetic discrimination. Such progressive changes may motivate more carriers of expanded alleles and unascertained symptomatic individuals to participate in genetic and intervention studies. Increased participation will facilitate larger genetic studies and clinical trials, which are crucial for the successful development of effective treatments for this devastating disorder.

## Supplementary Material

fcaf194_Supplementary_Data

## Data Availability

The complete dataset for the exponential decay model is provided in Supplementary Table 1. Analysis R codes are described in the Supplementary material. Cohort-based *HTT* CAG frequency distributions were sourced from the gnomAD browser (v3.1.2; https://gnomad.broadinstitute.org/) and the All of Us data browser (downloaded 8 April 2022; https://www.researchallofus.org/data-tools/). Validation data were obtained from the UK Biobank (https://www.ukbiobank.ac.uk/). Population pyramid data for the All of Us were obtained from https://www.researchallofus.org/data-tools/data-snapshots/. The population pyramid structure of the EU was retrieved from https://www.populationpyramid.net/europe/2020/. Analysis code is available from the corresponding author upon reasonable request.

## References

[1] HDCRG . A novel gene containing a trinucleotide repeat that is expanded and unstable on Huntington’s disease chromosomes. The Huntington’s Disease Collaborative Research Group. Cell. 26 1993;72(6):971–983. 8458085 10.1016/0092-8674(93)90585-e

[2] Bates GP, Dorsey R, Gusella JF, et al Huntington disease. Nat Rev Dis Primers. 2015;1:15005. 27188817 10.1038/nrdp.2015.5

[3] Orr HT, Zoghbi HY. Trinucleotide repeat disorders. Annu Rev Neurosci. 2007;30:575–621. 17417937 10.1146/annurev.neuro.29.051605.113042

[4] Pearson CE, Nichol Edamura K, Cleary JD. Repeat instability: Mechanisms of dynamic mutations. Nat Rev Genet. 2005;6(10):729–742. 16205713 10.1038/nrg1689

[5] GeM-HD Consortium . CAG repeat not polyglutamine length determines timing of Huntington’s disease onset. Cells. 2019;178(4):887–900.e14. 10.1016/j.cell.2019.06.036 PMC6700281 31398342

[6] Kay C, Collins JA, Wright GEB, et al The molecular epidemiology of Huntington disease is related to intermediate allele frequency and haplotype in the general population. Am J Med Genet B Neuropsychiatr Genet. 2018;177(3):346–357. 29460498 10.1002/ajmg.b.32618

[7] Kokmen E, Ozekmekci FS, Beard CM, O'Brien PC, Kurland LT. Incidence and prevalence of Huntington’s disease in Olmsted County, Minnesota (1950 through 1989). Arch Neurol. 1994;51(7):696–698. 8018043 10.1001/archneur.1994.00540190076018

[8] Pringsheim T, Wiltshire K, Day L, Dykeman J, Steeves T, Jette N. The incidence and prevalence of Huntington’s disease: A systematic review and meta-analysis. Mov Disord. 2012;27(9):1083–1091. 22692795 10.1002/mds.25075

[9] Shokeir MH . Investigation on Huntington’s disease in the Canadian Prairies. II. Fecundity and fitness. Clin Genet. 1975;7(4):349–353. 123838 10.1111/j.1399-0004.1975.tb00341.x

[10] Rawlins MD, Wexler NS, Wexler AR, et al The prevalence of Huntington’s disease. Neuroepidemiology. 2016;46(2):144–153. 26824438 10.1159/000443738

[11] Evans SJ, Douglas I, Rawlins MD, Wexler NS, Tabrizi SJ, Smeeth L. Prevalence of adult Huntington’s disease in the UK based on diagnoses recorded in general practice records. J Neurol Neurosurg Psychiatry. 2013;84(10):1156–1160. 23482661 10.1136/jnnp-2012-304636 PMC3786631

[12] Spinney L . Uncovering the true prevalence of Huntington’s disease. Lancet Neurol. 2010;9(8):760–761. 20594915 10.1016/S1474-4422(10)70160-5

[13] Falush D, Almqvist EW, Brinkmann RR, Iwasa Y, Hayden MR. Measurement of mutational flow implies both a high new-mutation rate for Huntington disease and substantial underascertainment of late-onset cases. Am J Hum Genet. 2001;68(2):373–385. 11225602 10.1086/318193 PMC1235271

[14] Lee JM, Ramos EM, Lee JH, et al CAG repeat expansion in Huntington disease determines age at onset in a fully dominant fashion. Neurology. 2012;78(10):690–695. 22323755 10.1212/WNL.0b013e318249f683 PMC3306163

[15] GeM-HD Consortium . Identification of genetic factors that modify clinical onset of Huntington’s disease. Cell. 2015;162(3):516–526. 26232222 10.1016/j.cell.2015.07.003 PMC4524551

[16] Dolzhenko E, Deshpande V, Schlesinger F, et al ExpansionHunter: A sequence-graph-based tool to analyze variation in short tandem repeat regions. Bioinformatics. 2019;35(22):4754–4756. 31134279 10.1093/bioinformatics/btz431 PMC6853681

[17] Rawlins M . Huntington’s disease out of the closet? Lancet. 2010;376(9750):1372–1373. 20594589 10.1016/S0140-6736(10)60974-9

[18] Keum JW, Shin A, Gillis T, et al The HTT CAG-expansion mutation determines age at death but not disease duration in Huntington disease. Am J Hum Genet. 2016;98(2):287–298. 26849111 10.1016/j.ajhg.2015.12.018 PMC4746370

[19] Langbehn DR, Brinkman RR, Falush D, Paulsen JS, Hayden MR; International Huntington's Disease Collaborative G. A new model for prediction of the age of onset and penetrance for Huntington’s disease based on CAG length. Clin Genet. 2004;65(4):267–277. 15025718 10.1111/j.1399-0004.2004.00241.x

[20] Apolinario TA, da Silva IDS, Agostinho LA, Paiva CLA. Investigation of intermediate CAG alleles of the HTT in the general population of Rio de Janeiro, Brazil, in comparison with a sample of Huntington disease-affected families. Mol Genet Genomic Med. 2020;8(4):e1181. 32067426 10.1002/mgg3.1181 PMC7196456

[21] Costa MDC, Magalhaes P, Guimaraes L, Maciel P, Sequeiros J, Sousa A. The CAG repeat at the Huntington disease gene in the Portuguese population: Insights into its dynamics and to the origin of the mutation. J Hum Genet. 2006;51(3):189–195. 16372132 10.1007/s10038-005-0343-8

[22] Semaka A, Kay C, Doty CN, Collins JA, Tam N, Hayden MR. High frequency of intermediate alleles on Huntington disease-associated haplotypes in British Columbia’s general population. Am J Med Genet B Neuropsychiatr Genet. 2013;162B(8):864–871. 24038799 10.1002/ajmg.b.32193

[23] Brayne C, Moffitt TE. The limitations of large-scale volunteer databases to address inequalities and global challenges in health and aging. Nat Aging. 2022;2(9):775–783. 37118500 10.1038/s43587-022-00277-x PMC10154032

[24] Fry A, Littlejohns TJ, Sudlow C, et al Comparison of sociodemographic and health-related characteristics of UK Biobank participants with those of the general population. Am J Epidemiol. 2017;186(9):1026–1034. 28641372 10.1093/aje/kwx246 PMC5860371

[25] Rubinsztein DC, Amos W, Leggo J, et al Mutational bias provides a model for the evolution of Huntington’s disease and predicts a general increase in disease prevalence. Nat Genet. 1994;7(4):525–530. 7951324 10.1038/ng0894-525

[26] Ibanez K, Jadhav B, Zanovello M, et al Increased frequency of repeat expansion mutations across different populations. Nat Med. 2024;30(11):3357–3368. 39354197 10.1038/s41591-024-03190-5 PMC11564083

[27] Kay C, Collins JA, Miedzybrodzka Z, et al Huntington disease reduced penetrance alleles occur at high frequency in the general population. Neurology. 2016;87(3):282–288. 27335115 10.1212/WNL.0000000000002858 PMC4955276

[28] Rubinsztein DC, Leggo J, Coles R, et al Phenotypic characterization of individuals with 30–40 CAG repeats in the Huntington disease (HD) gene reveals HD cases with 36 repeats and apparently normal elderly individuals with 36–39 repeats. Am J Hum Genet. 1996;59(1):16–22. 8659522 PMC1915122

[29] Fisher ER, Hayden MR. Multisource ascertainment of Huntington disease in Canada: Prevalence and population at risk. Mov Disord. 2014;29(1):105–114. 24151181 10.1002/mds.25717

[30] Penziner E, Williams JK, Erwin C, et al Perceptions of discrimination among persons who have undergone predictive testing for Huntington’s disease. Am J Med Genet B Neuropsychiatr Genet. 2008;147(3):320–325. 17948904 10.1002/ajmg.b.30600 PMC3645880

[31] Bombard Y, Veenstra G, Friedman JM, et al Perceptions of genetic discrimination among people at risk for Huntington’s disease: A cross sectional survey. Bmj. 2009;338:b2175. 19509425 10.1136/bmj.b2175 PMC2694258

[32] Petracca M, Di Tella S, Solito M, et al Clinical and genetic characteristics of late-onset Huntington’s disease in a large European cohort. Eur J Neurol. 2022;29(7):1940–1951. 35357736 10.1111/ene.15340 PMC9324106

[33] James CM, Houlihan GD, Snell RG, Cheadle JP, Harper PS. Late-onset Huntington’s disease: A clinical and molecular study. Age Ageing. 1994;23(6):445–448. 9231935 10.1093/ageing/23.6.445

[34] Lipe H, Bird T. Late onset Huntington disease: Clinical and genetic characteristics of 34 cases. J Neurol Sci. 2009;276(1–2):159–162. 18977004 10.1016/j.jns.2008.09.029 PMC3140172

[35] Duff K, Paulsen JS, Beglinger LJ, Langbehn DR, Stout JC, Predict HDIotHSG. Psychiatric symptoms in Huntington’s disease before diagnosis: The predict-HD study. Biol Psychiatry. 2007;62(12):1341–1346. 17481592 10.1016/j.biopsych.2006.11.034

[36] Paoli RA, Botturi A, Ciammola A, et al Neuropsychiatric burden in Huntington’s disease. Brain Sci. 2017;7(6):67. 28621715 10.3390/brainsci7060067 PMC5483640

[37] McAllister B, Gusella JF, Landwehrmeyer GB, et al Timing and impact of psychiatric, cognitive, and motor abnormalities in Huntington disease. Neurology. 2021;96(19):e2395–e2406. 33766994 10.1212/WNL.0000000000011893 PMC8166441

[38] Kremer B, Almqvist E, Theilmann J, et al Sex-dependent mechanisms for expansions and contractions of the CAG repeat on affected Huntington disease chromosomes. Am J Hum Genet. 1995;57(2):343–350. 7668260 PMC1801544

[39] Leeflang EP, Tavare S, Marjoram P, et al Analysis of germline mutation spectra at the Huntington’s disease locus supports a mitotic mutation mechanism. Hum Mol Genet. 1999;8(2):173–183. 9931325 10.1093/hmg/8.2.173

[40] Goldberg YP, Kremer B, Andrew SE, et al Molecular analysis of new mutations for Huntington’s disease: Intermediate alleles and sex of origin effects. Nat Genet. 1993;5(2):174–179. 8252043 10.1038/ng1093-174

[41] Dewan R, Chia R, Ding J, et al Pathogenic huntingtin repeat expansions in patients with frontotemporal dementia and amyotrophic lateral sclerosis. Neuron. 2021;109(3):448–460 e4. 33242422 10.1016/j.neuron.2020.11.005 PMC7864894

[42] Perlis RH, Smoller JW, Mysore J, et al Prevalence of incompletely penetrant Huntington’s disease alleles among individuals with major depressive disorder. Am J Psychiatry. 2010;167(5):574–579. 20360314 10.1176/appi.ajp.2009.09070973 PMC3114558

