# Supplementary material for: Significant underascertainment in Huntington’s disease
Source: Brain Commun. 2025 May 20;7(3):fcaf194. doi: 10.1093/braincomms/fcaf194 (PMC12123046; doi:10.1093/braincomms/fcaf194)
Supplement: fcaf194_Supplementary_Data [file fcaf194_supplementary_data.doc]

**Supplementary Table 1: Frequencies of CAG repeats of the study samples.**

In this study, we analyzed 7,578 unrelated HD subjects of European ancestry, previously included in our HD modifier GWAS, to develop statistical models to infer the frequencies of expanded CAG repeats in the general population. The percentage frequencies of each CAG repeat size are summarized based on the total number of alleles (n=15,156).

| CAG | Count | Percent |  | CAG | Count | Percent |
| --- | --- | --- | --- | --- | --- | --- |
| 8 | 1 | 0.01 |  | 32 | 13 | 0.09 |
| 9 | 52 | 0.34 |  | 33 | 8 | 0.05 |
| 10 | 49 | 0.32 |  | 34 | 6 | 0.04 |
| 11 | 2 | 0.01 |  | 35 | 6 | 0.04 |
| 12 | 53 | 0.35 |  | 36 | 6 | 0.04 |
| 13 | 11 | 0.07 |  | 37 | 3 | 0.02 |
| 14 | 59 | 0.39 |  | 38 | 37 | 0.24 |
| 15 | 676 | 4.46 |  | 39 | 131 | 0.86 |
| 16 | 382 | 2.52 |  | 40 | 513 | 3.38 |
| 17 | 2592 | 17.1 |  | 41 | 1038 | 6.85 |
| 18 | 1093 | 7.21 |  | 42 | 1191 | 7.86 |
| 19 | 650 | 4.29 |  | 43 | 1166 | 7.69 |
| 20 | 552 | 3.64 |  | 44 | 973 | 6.42 |
| 21 | 285 | 1.88 |  | 45 | 694 | 4.58 |
| 22 | 247 | 1.63 |  | 46 | 518 | 3.42 |
| 23 | 244 | 1.61 |  | 47 | 391 | 2.58 |
| 24 | 214 | 1.41 |  | 48 | 261 | 1.72 |
| 25 | 104 | 0.69 |  | 49 | 207 | 1.37 |
| 26 | 95 | 0.63 |  | 50 | 139 | 0.92 |
| 27 | 65 | 0.43 |  | 51 | 101 | 0.67 |
| 28 | 45 | 0.3 |  | 52 | 72 | 0.48 |
| 29 | 37 | 0.24 |  | 53 | 58 | 0.38 |
| 30 | 17 | 0.11 |  | 54 | 45 | 0.3 |
| 31 | 20 | 0.13 |  | 55 | 34 | 0.22 |

**Supplementary Table 2: Comparison of age-adjusted exponential decay model with literature.**

The frequencies of intermediate, reduced penetrance, fully penetrant, and all pathogenic repeats in the general population were obtained from our age-adjusted exponential model. Subsequently, these estimates were compared to frequencies of repeats reported in the literature.

& denotes the data from Table S7, EUR, 100K GP project.

| CAG repeat range | Allele frequency | | | | |
| --- | --- | --- | --- | --- | --- |
|  | Age-adjusted exponential decay model | Semaka et. al.[^1^](#_ENREF_1) | Kay et. al.[^2^](#_ENREF_2) | Gardiner et. al.[^3^](#_ENREF_3) | Ibanez et. al.[^4^](#_ENREF_4) ^&^ |
| Intermediate  (27-35 CAGs) | 2.86%  (one in 17 people) | 2.92%  (one in 17 people) | 3.13%  (one in 16 people) | 3.2%  (one in 16 people) | NA |
| Reduced penetrant  (36-39 CAGs) | 0.12%  (one in 417 people) | 0.19%  (one in 263 people) | 0.103%  (one in 485 people) | 0.058%  (one in 862 people) | 0.0932%  (one in 536 people) |
| Fully penetrant  (>39 CAGs) | 0.0337%  (one in 1,484 people) | NA | 0.021%  (one in 2,381 people) | NA | 0.022%  (one in 2,269 people) |
| All pathogenic  (>35 CAGs) | 0.1537%  (one in 325 people) | NA | 0.123%  (one in 407 people) | NA | NA |

**Supplementary Figure 1: Overall flow of the study.**

To estimate the prevalence of expanded *HTT* CAG repeats in the general population, we developed an exponential decay model based on the frequencies of unexpanded repeats and extended it to the expanded repeat range. Next, we adjusted the population frequencies of expanded repeats for age using an HD survival model, accounting for decreased survival in HD. Then, we applied the HD onset probability to the age-adjusted frequencies of expanded repeats to estimate the proportion of individuals carrying expanded repeats who have developed symptoms. Finally, we compared the population frequencies of symptomatic HD individuals to the reported disease prevalence to estimate the level of clinical ascertainment in HD.

**Supplementary Figure 2: Unexpanded CAG repeats in HD subjects represent the unexpanded repeats in the population samples.**

To assess whether the distribution of repeat lengths in the unexpanded repeats of HD subjects reflects that of the general population, we compared the percent frequency of CAG repeats smaller than 36 (black; n=7,578 unexpanded repeats) to those observed in gnomAD (Europeans; purple), All of Us (Europeans; cyan), and UK Biobank (all populations, gold). The y-axis represents the percent frequency of each repeat.

**Supplementary Figure 3: Exponential decrease of frequency continues beyond 35 CAGs.**

To assess the exponential decrease in frequency above the most common repeat size, we evaluated the linearity of log10-transformed repeat frequencies (y-axis) in gnomAD (Europeans; purple line; n>110,831 alleles for each repeat), All of Us (Europeans; cyan line; n=425,906 alleles for each repeat), and the UK Biobank (all populations; gold line; n=980,760 alleles in total). Y-axis and x-axis represent % frequency in log scale and CAG repeat size, respectively.

**Supplementary Figure 4: Fitting statistical distributions to CAG repeat frequency.**

Commonly used probability distributions were fitted to the observed frequency distribution of unexpanded CAG repeats in clinically ascertained HD subjects (histogram; n=7,578 unexpanded repeats). The fitted distributions are represented by different colors: normal (red), Poisson (blue), Cauchy (purple), gamma (orange), log-normal (yellow), logistic (grey), and Weibull (cyan). The y-axis shows frequency in density format, while the x-axis represents CAG repeat size.

**
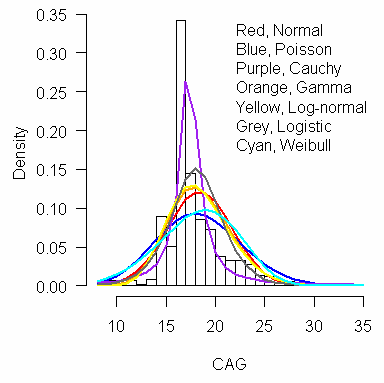
**

**Supplementary Figure 5: Decreased survival probability in HD compared to the US general population.**

The survival curves for HD subjects (red) and the general population (black) were derived from HD subjects with recorded ages at death (n = 1,016; 36-120 CAG repeats) and age-specific death probabilities from the US Social Security Administration actuarial life table, respectively. For HD, the percentage survival was calculated by dividing the number of deaths at a given age by the total number of recorded deaths.

**Supplementary Figure 6: CAG-specific parametric survival model for HD.**

To refine our original exponential decay model, we developed a survival probability curve for each CAG repeat length through parametric survival analysis. Due to limited sample size, our analysis focused on CAG repeat lengths of 40-50, which represent approximately 90% of our ascertained samples. For each CAG repeat length, we constructed a parametric survival model using a lognormal distribution (red line), which is overlaid on the observed survival curve (black line) for visual comparison. A black triangle in each panel marks the age at which 50% survival is estimated according to the survival model.

**Supplementary Figure 7: Age distributions of population samples.**

Simulation data were generated based on the age distribution of each population dataset, including All of Us (A; percent data were obtained from https://www.researchallofus.org/data-tools/data-snapshots/) and UK Biobank (B; n=502,618). Frequency was based on male and female combined data. The y-axis represents the percent frequency of expanded CAG repeats, while the x-axis represents age.

**Supplementary Figure 8: CAG-specific parametric survival model for HD onset.**

To calculate the proportion of HD subjects with clinical onset at a given time point, we constructed a parametric survival model for onset for each CAG repeat length. Focusing on the 40-50 CAG range, we employed a lognormal distribution to create the parametric survival probability model (blue line), which was then overlaid on the observed onset survival curve (black line) to evaluate the levels of fitting. A black triangle in each panel indicates the age at which 50% of individuals are expected to experience clinical onset, as determined by the onset survival model.

**Supplementary Figure 9: Estimating the proportion of ascertainable expanded repeat carriers for each CAG.**

To calculate the frequency of expanded repeat carriers with disease symptoms in the general population, we modified our age-adjusted exponential decay model using onset survival data. For each specific CAG repeat length, the plots display the frequencies of expanded repeats in black based on our age-adjusted exponential decay model, while the frequencies of individuals carrying expanded repeats with disease symptoms are shown in blue (based on the combined sample size, n=8,608 for survival model).

**Supplementary Figure 10: Age distribution of EU.**

Simulation data were generated based on the age distribution of the European Union (EU). Percent frequency data were obtained from https://www.populationpyramid.net/europe/2020/. Frequency was based on male and female combined data (based on n=749,524,044). The y-axis represents the percent frequency of expanded CAG repeats, while the x-axis represents age.

**Analysis codes**

# Reconstruct the repeats data using S.Table 1

cag1 = rep ("expanded.cag", by='count.expanded.cag')

cag2.cag = rep ("unexpanded.cag", by='count.unexpanded.cag')

mutant = 7578 ; all=mutant*16128; normal = all - mutant

mutant.cag = cag1 ; normal.cag = sample(cag2, normal, replace = T)

# bootstrapping

all.cag = c(mutant.cag, normal.cag)

# Repeat distribution figure

hist(all.cag, main = '', br = 100, xlab = 'CAG')

hist(all.cag, main = '', br = 100, xlab = 'CAG', ylim = c(0, 100000))

hist(all.cag, main = '', br = 100, xlab = 'CAG', ylim = c(0, 3000))

# Make count data

final = as.data.frame(table(all.cag))

# Split count data into normal and expanded repeat

normal = final[which(final$cag < 36), ] ; normal

mutant = final[which(final$cag > 35), ] ; mutant

# Model using bootstrapped normal repeat

mod = lm(log.count ~ cag , data = normal) ; summary(mod)

# prediction for the mutant

x = 36:55 ; y = predict(mod, list(cag = x))

pred= data.frame(cag = x, log.count = y)

pred$count = 10 ^ pred$log.count

# prediction for the normal

x = 17:35 ; y = predict(mod, list(cag = x))

pred.normal = data.frame(cag = x, log.count = y)

pred.normal$count = 10 ^ pred.normal$log.count

# plot prediction and observed

plot(normal$cag, normal$log.count , main = '', xlab = 'CAG', ylab = 'Log10(Frequency)' , typ = 's', xlim = c(17, 55), ylim = c(0, 8), col = 'blue', lwd = 2)

abline(mod, lwd = my.lwd, col = 'gray', lty=2)

points(mutant$cag, mutant$log.count , typ = 's', col = 2, lwd = my.lwd )

# points(pred$cag, pred$log.count , typ = 's', col = 1, lwd = my.lwd )

points(normal$cag, normal$log.count , typ = 's', col = 'blue', lwd = my.lwd )

# Fitting to known distributions

# Start with reconstructed repeat data

library(MASS)

hist.data = hist(cag2, freq=F, main="", xlab="CAG", br=26)

# Distribution of normal repepat

hist(cag2, freq=F, main="", xlab="CAG", br=26)

# Normal Distribution

fitdistr(cag2, "normal")

xpos=seq(8, 35, 1)

ypos=dnorm(xpos, mean(cag2), sd(cag2))

points(xpos, ypos, type="l", col=2, lwd=2)

# Poisson Distribution

fitdistr(cag2,"poisson")

ypos=dpois(xpos, mean(cag2))

points(xpos, ypos, type="l", col='blue', lwd=2)

# Negative Binomial Distribution: do not use

fitdistr(cag2, "negative binomial")

# Cauchy Distribution

fitdistr(cag2, "cauchy")

ypos=dcauchy(xpos, location=17.34761967 , scale=1.10056122)

points(xpos, ypos, type="l", col='purple', lwd=2)

# Exponential Distribution: do not use

cag2.right = cag2 [which (cag2> 16)]

fitdistr(cag2.right, "exponential")

# Gamma Distribution

fitdistr(cag2, "gamma")

ypos=dgamma(xpos, shape= 33.59131048 , rate=1.82447947)

points(xpos, ypos, type="l", col='orange', lwd=2)

# Log-Normal Distribution

fitdistr(cag2, "log-normal")

ypos=dlnorm(xpos, meanlog= 2.898014044 , sdlog=0.172004511)

points(xpos, ypos, type="l", col='yellow', lwd=2.5)

# Logistic Distribution

fitdistr(cag2, "logistic")

ypos=dlogis(xpos, location=18.04217096 , scale=1.64861768)

points(xpos, ypos, type="l", col='dimgrey', lwd=2)

# Weibull Distribution

fitdistr(cag2, "weibull")

ypos=dweibull(xpos, shape= 5.17082216 , scale=19.81236741)

points(xpos, ypos, type="l", col='cyan', lwd=2)

# legend

legend('topright', c('Red, Normal', 'Blue, Poisson', 'Purple, Cauchy', 'Orange, Gamma', 'Yellow, Log-normal', 'Grey,

Logistic', 'Cyan, Weibull'), bty = 'n', cex=1)

# Age adjustment. Continue from the previous session.

library(survival)

# US survival: Read in US survival data from <https://www.ssa.gov/oact/STATS/table4c6.html>

cag.range = 40:50
freq = c(1.26474E-02, 9.17792E-03, 6.66019E-03, 4.83313E-03, 3.50728E-03, 2.54514E-03, 1.84694E-03, 1.34028E-03, 9.72608E-04, 7.05797E-04, 5.12179E-04)

# Begin plotting

plot(1, type="n", main='', xlab='Age (years)', ylab='Age-adjusted frequency (%)', xlim = c(-10, 100), ylim=c(0, 0.013))

pred.age = seq(0, 100, by = 1)

combined = pred.age

# Add for each cag

for ( i in 1:11) {

cag = cag.range [i]

# Survival model based on lognrmal distribution

sub = het.ad [ which (het.ad$cag1 == cag), ] ; dim(sub) ; head(sub)

sub2 = sub [, c(10, 15)] ; names(sub2) = c('cag', 'age') ; sub2$status = rep(1, nrow(sub2))

mod = survreg(Surv(age, status) ~ 1, data = sub2, dist='lognormal')

new.data = data.frame(cag = rep(i, length(pred.age)))

hd.surv.prob = 1 - pnorm((log(pred.age) - predict(mod, new.data, type = "lp")) / mod$scale)

res = data.frame (age = pred.age, hd.surv.prob) ; head(res)

res$us.surv.prob = us$survival/100 ; head(res)

res$total.surv.prob = res$hd.surv.prob * res$us.surv.prob

# To calculate freqeuency directly

res$freq.adj = freq[i] * res$total.surv.prob ; head(res)

lines(res$age, res$freq.adj, col = 'black', lwd=2)

# To calculate count based - 100K people, 200K chromosomes

hd = (freq[i]/100)*200000 * res$total.surv.prob

gen = 200000

res$freq.adj = hd/gen*100 ; lines(res$age, res$freq.adj, col = 'black', lwd=2)

}

**Supplemental Reference**

1. Semaka A, Kay C, Doty CN, Collins JA, Tam N, Hayden MR. High frequency of intermediate alleles on Huntington disease-associated haplotypes in British Columbia's general population. *American journal of medical genetics Part B, Neuropsychiatric genetics : the official publication of the International Society of Psychiatric Genetics*. Dec 2013;162B(8):864-71. doi:10.1002/ajmg.b.32193

2. Kay C, Collins JA, Miedzybrodzka Z*, et al*. Huntington disease reduced penetrance alleles occur at high frequency in the general population. *Neurology*. Jul 19 2016;87(3):282-8. doi:10.1212/WNL.0000000000002858

3. Gardiner SL, Boogaard MW, Trompet S*, et al*. Prevalence of Carriers of Intermediate and Pathological Polyglutamine Disease-Associated Alleles Among Large Population-Based Cohorts. *JAMA neurology*. Jun 1 2019;76(6):650-656. doi:10.1001/jamaneurol.2019.0423

4. Ibanez K, Jadhav B, Zanovello M*, et al*. Increased frequency of repeat expansion mutations across different populations. *medRxiv : the preprint server for health sciences*. Jul 8 2024;doi:10.1101/2023.07.03.23292162
